# Monteggia fracture with unreducable anterior dislocation of the radial head and a lesion of the external collateral ligament of the elbow

**DOI:** 10.11604/pamj.2018.29.216.12996

**Published:** 2018-04-18

**Authors:** Aymen Saidi, Lassaad Hassini, Youcef Othmen, Aymen Fekih, Mohamed Allagui, Issam Aloui, Abderrazek Abid

**Affiliations:** 1 Department of Orthopaedic Surgery, University Hospital, Monastir 5000, Tunisia

**Keywords:** Monteggia-fracture, unreducable, anterior dislocation

## Abstract

Monteggia described a fracture of the proximal third of the ulna with anterior dislocation of the radial head from both the proximal radioulnar and radiocapitellar joints. The key treatment principle in Monteggia fractures is stable anatomic alignment of the ulna. We present an uncommon case of a Monteggia fracture-dislocation with an unreducable anterior dislocation of the radial head and associated with a lesion of the lateral collateral ligament of the elbow. The patient in our report had a successful clinical outcome and functional range of motion after rigid fixation of the ulnar shaft fracture and exploration of the elbow joint, reduction of the radial head and repair of the lateral collateral ligament. This case is unusual because of the association of a complete tear of the external collateral ligament of the elbow.

## Introduction

The original fracture pattern described by Monteggia is a fracture of the proximal third of the ulna with anterior dislocation of the radial head. Proximal radius was dislocated from both the proximal radioulnar (RU) and radiocapitellar (RC) joints. The main step in the surgical treatment of Monteggia fractures is anatomic reduction and stable osteosynthesis. Once this step is properly achieved, radial head is automatically reduced in most of the cases. Open reduction is only necessary in the rare cases of incarceration of soft parts or a bone fragment. We report a case of a Monteggia fracture were open reduction of the radial head was performed. This case is unusual because of the association of a complete tear of the external collateral ligament of the elbow.

## Patient and observation

**Figure 1:**
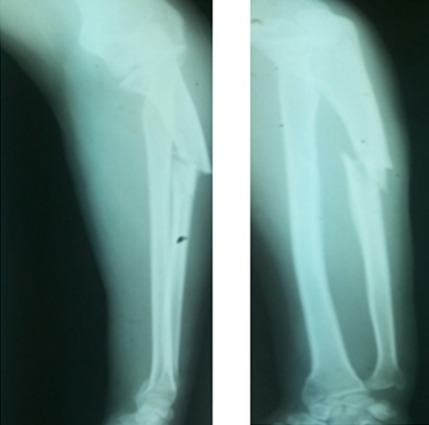
Plain radiographs of left forearm, showing a dislocation of the radial head and a segmental displaced ulna shaft fracture (Monteggia fracture-dislocation type 1) associated with a radial styloid fracture

**Figure 2:**
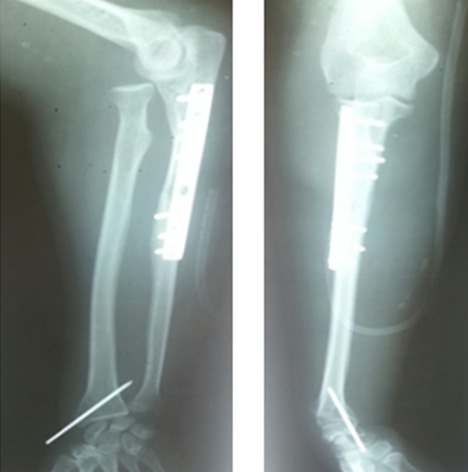
Postoperative radiographs showing a good reduction of the fracture but persistent anterior dislocation of the radial head

**Figure 3:**
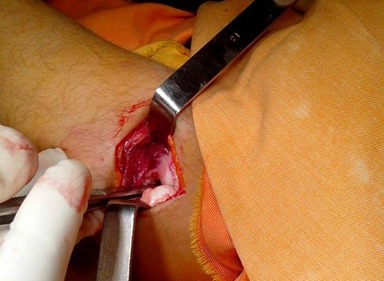
Peroperative photo showing anterior dislocation of the radial head and complete tear of lateral collateral ligament

**Figure 4:**
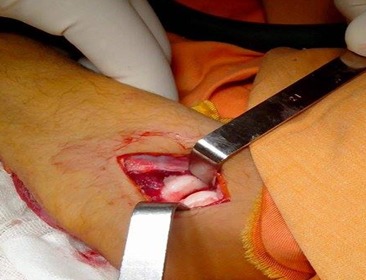
The radial head was reduced and the lateral collateral ligament was repaired

**Figure 5:**
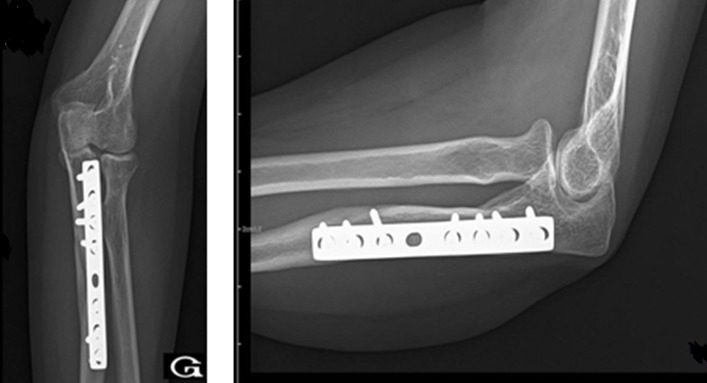
Satisfactory range of motion 2 years later

**Figure 6:**
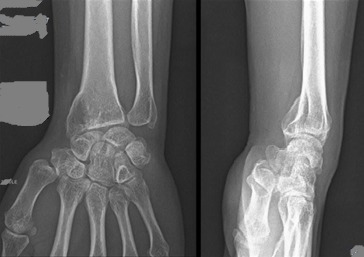
Radiographs obtained 2 years later showing an anatomical position of the radial head and the fracture was healed in good alignment

**Figure 7:**
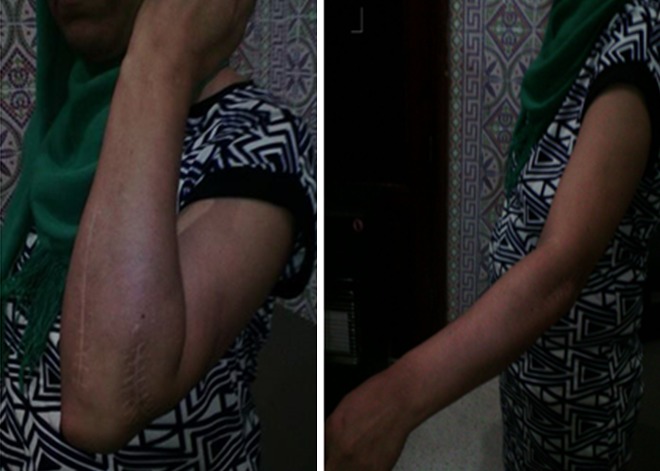
Radiographs obtained 2 years later showing a consolidated fracture of the radial styloid

A 42-year-old patient presents in the emergency ward with a fresh closed trauma from the left forearm, following a road accident. Physical examination showed swelling of the elbow and upper forearm and an obvious deformity. The patient was neuro-vascularly intact in that extremity. Initial plain radiographic studies of the left forearm revealed a displaced fracture involving the ulna shaft and a dislocation of the radial head. This radiological finding, also called Monteggia fracture-dislocation type 1, was associated with a radial styloid fracture (Figure 1). Posterior approach was used and Ulna shaft fracture was reduced and fixed with an 8-hole 3.5 mm compression plate. The radial styloid fracture was stabilized by a pin. Postoperative radiography in the operating room showed a good reduction of the Ulna fracture but persistent anterior dislocation of the radial head (Figure 2). Next the elbow joint was approached through a separate lateral incision. We revealed a radial head which had buttonholed through the capsule, associated with a ruptured annular ligament (Figure 3). The radial head was reduced and then the lateral collateral ligament was repaired (Figure 4). Postoperatively the limb was immobilized in a splint at 90° of flexion and she was started on gentle range of motion exercises post-operatively. At 2 years follow up, the patient have a satisfactory range of motion (Figure 5) and the radiographs showed an anatomical position of the radial head and the fractures were consolidated in good alignment (Figure 6, Figure 7).

## Discussion

In 1814 Monteggia reported a particular injury pattern associating a fracture of the proximal third of the shaft of the ulna, with a dislocation of the radial head from both the superior radio-ulnar and the radio-humeral joints [1-3]. Dislocation could present in an anterior a posterior variety. This injury pattern represents 2-5% of proximal forearm fractures [4] and 0.7% of all elbow fractures and dislocations in adult population [2]. Multiple classification systems of the Monteggia fracture exist. The one that is universally used is Bado's classification. This classification system defines four types according to of the direction of the dislocation of the radial head and the angulation of the ulna [2]. Type I is an anterior dislocation of the radial head and proximal anterior angulation of the ulnar fracture. Type II is a posterior or postero-lateral dislocation of the radial head with a proximal posterior angulation of the ulnar fracture. Type III is an anterolateral dislocation of the radial head with a metaphyseal ulnar fracture. Type IV is an anterior dislocation of the radial head with a proximal radial and ulnar shaft fracture in the same level. A modification of this classification by Dormans and Ranglater added a fifth type. It is a fracture of the radius with a dislocation of the proximal radio-ulnar and the radio-capitellar joints [1]. The Monteggia fracture in adults always requires a surgical treatment [2]. An anatomical reduction and a stable osteosynthesis through a posterior approach of the ulna is the key step of this surgery. Radial head is generally automatically reduced after this step. According to Soubeyrand et al [5], a proximal ulnar-radio dislocation in the case of a Monteggia-type fracture will be automatically reduced when the fracture of the ulna is reduced. The anatomic reduction of the ulna determines the reduction of the radial head spontaneously in 93% of the cases. In the other 7%, open reduction typically needs to be performed [2]. A perfect alignment of the ulna fracture before osteosynthesis is necessary in order to obtain the RC joint reduction. In opposition to most other diaphyseal fractures where one could tolerate a certain degree of imperfection in reduction and then intramedullary nailing is an interesting option, perfect restoration of the anatomy of the ulna with and open plate osteosynthesis is mandatory is the context of Monteggia fracture [5]. During the operation and in the later follow-up, careful clinical and radiological evaluation of the elbow is necessary in order to recognize residual instability of the RC joint. This could be caused by a loss of the ulnar fracture reduction [6, 7]. In a chronic context, revising the ulnar osteosynthesis is not enough is most of the case and open reduction and repairing the annular ligament are often required [1]. In fresh injuries, it is generally not be necessary to approach the RC joint except in the rare cases of incarceration of soft parts (annular ligament) or a bone fragment (fracture of the radial head). Capsule, annular ligament and biceps tendon were reported to be responsible for the irreducibility of the RC joint in Monteggia fractures [8, 9]. Morris [10] described an irreducible dislocation of the radial head caused by the entrapment of the posterior interosseous nerve between the radial head and the ulna. In front of an irreducibility of the dislocation the surgeon should recheck the quality of the ulnar fracture alignment and then verify the presence of interposed bony or soft tissue fragments. According to Reynders et al [3], open reduction of the radial head with reconstruction of the annular ligament could be responsible of a proximal synostosis between radius and ulna. Avoiding simultaneous exposure of both bones of the forearm and of early active mobilization could reduce the risk of this complication.

## Conclusion

we have described the management of a rare lesion in which there was buttonholing of the radial head through the anterior capsule associated with a rupture and incarceration of the annular ligament, causing the radiocapitellar dislocation to be irreducible (even after fixation of the ulnar fractures). The anatomic reduction of the ulna determines the reduction of the radial head spontaneously in 93% of the cases. In the other 7%, open reduction typically needs to be performed.

## Competing interests

The authors declare no competing interests.
